# Supporting Healthful Eating Through Retail Environmental Change: Communities Putting Prevention to Work

**DOI:** 10.5888/pcd10.130166

**Published:** 2013-11-14

**Authors:** Latetia V. Moore

More healthful eating patterns can help people achieve recommended nutrient intakes, control calorie intake, and reduce the risk of some of the leading causes of chronic illness and death in the United States such as cardiovascular disease, diabetes, and certain types of cancer (1). Making more healthful choices easily accessible may help people start and maintain more healthful eating patterns (2,3). Although research and programs dedicated to improving access to more healthful food choices are under way (4), there is little empirical evidence to identify what aspects of the retail environment require intervention to improve access (5). It is unclear whether efforts should focus on improving proximity to stores, improving the selection of healthful options within stores with limited stock, making more healthful items more affordable, improving the quality of goods within stores, introducing more healthful options to an area via other retail models like farmers markets, or some combination of the above. One recent initiative that may elucidate what aspects of the environments to change to improve the diet and health of community members is Communities Putting Prevention to Work (CPPW). During the 2-year funding period beginning in 2010, 39 of the 50 CPPW communities committed to enhancing access to healthful food and evaluating the effect of this increased access on behavior (6). CPPW presented an opportunity to evaluate changes to the environment that may support people in making more healthful dietary choices.

This collection of articles in *Preventing Chronic Disease* presents findings from the first 7 of those 39 communities to publish results from their CPPW retail food environment initiatives. Three communities focused their efforts on improving access in corner stores. The other 4 communities chose to improve access at farmers markets in low-income areas via incentive programs or increased acceptance of food assistance benefits.

Improving access does not necessarily mean adding large, full-service grocery stores like supermarkets and supercenters to areas with low levels of access. Although these types of stores frequently offer a wider selection of high-quality, affordable, and more healthful options (2), new store development may not always be possible (7). Smaller retail venues such as small grocery stores, corner stores, and fruit and vegetable markets can also play a role in ensuring communities have adequate access to more healthful foods (8).

Each of 3 highlighted CPPW initiatives working to improve access via corner stores used qualitative or quantitative baseline assessments to identify potential barriers, engage community residents, understand neighborhood context, and develop solutions to increase access. In Pitt County, North Carolina, store assessments indicated that the pricing and quality of food items in rural corner stores were similar to that of food items in urban corner stores, but the rural corner stores were more likely to carry healthful foods (9). Qualitative interview results indicated that corner store owners were willing to stock more healthful foods but perceived low customer demand for them in underserved areas, despite the fact that more than half of customers of these stores reported that a wider selection of groceries and better prices would help them buy more groceries at corner stores (10). The awardee used these findings to establish a baseline and to identify small stores that offered limited produce to target for a healthy corner store initiative. The initiative included introducing potentially profitable healthful food items, on the basis of store owner perceptions of demand and findings from customer surveys, and offering taste tests and in-store promotions to increase demand for fruits and vegetables.

Quantitative and qualitative baseline assessments by the second of the 3 CPPW awardees in Nashville, Tennessee, focused more narrowly on 4 areas researchers identified as providing limited access, based on existing data (11). Physical assessment of the stores in these areas indicated that few of them offered fresh fruits, fresh vegetables, low-fat milk, or whole-wheat bread and none stocked items from all 4 of these food categories (11). Qualitative assessments identified a neighborhood history of poor-quality produce offered in small stores, mistrust of store proprietors, and mistrust of government (11). As in North Carolina, the Tennessee awardee used this information as a baseline and to inform the design of a corner store initiative and communications campaign. Their efforts aimed to increase awareness of the higher-quality product offerings introduced by the initiative such as seasonal fresh fruits and vegetables purchased in bulk from a local mobile market. The initiative also developed a plan to build relationships between corner store proprietors and neighborhood organizations, such as a church, to mitigate mistrust. The third CPPW grantee in Cook County, Illinois, also identified and recruited corner stores into a healthy corner store initiative and engaged community members by conducting quantitative baseline assessments and partnering with 9 community institutions including local governments, community-based organizations, and faith-based institutions (12,13). Of the 53 corner stores approached, 25 corner stores were willing to add new healthful foods to their inventory, and 21 (84%) received new equipment and marketing materials and enhanced community outreach (13).

Introducing or altering inventory in corner stores is only one way of increasing access to more healthful food options. Farmers markets, farm stands, and other retail venues that sell fresh farm produce can increase access to high-quality fresh produce, be set up in a variety of locations, and be implemented at a low cost (14–16). They may also reduce the cost of healthful foods for low-income families and result in increased fruit and vegetable intake of program participants when federal food and nutrition assistance programs extend benefits to include farmers market purchases (15,16). In addition to the potential positive effect on diet of farmers markets and other retail venues that sell fresh farm produce, these retail venues may also help to bolster local economies and foster business growth and tourism (14).

Each of the 4 highlighted CPPW initiatives working to improve access at farmers markets in low-income areas was able to demonstrate that increasing affordability of fruits and vegetables through a bonus incentive program is a successful strategy for increasing Supplemental Nutrition Assistance Program (SNAP) use or sales or both at farmers markets. Two CPPW initiatives were also able to demonstrate increases in fruit and vegetable consumption. In 9 farmers markets in lower-income regions of King County, Washington, SNAP/EBT (electronic benefit transfer) acceptance rates increased by 79% for market stalls after introduction of subsidized (EBT) terminals for processing SNAP cards (17). Analyses of 4 years of EBT sales data in New York City showed that by the last 2 years, markets participating in the Health Bucks program had 87% to 98% higher daily EBT sales than markets without the incentive (18). In Pennsylvania, average SNAP sales more than doubled in the first 2 years of the Philly Food Bucks program (19). Philly Food Bucks users were also more than twice as likely to report increasing fruit and vegetable consumption and trying new fruits or vegetables as non-Philly Food Bucks users. The County of San Diego Health and Human Services Agency directly attributed 48% of the $1.7 million total market revenue in 5 farmers’ markets to an incentive program that offered matched monetary incentives of up to $20 per month (20). Participants at these markets also reported significant increases in daily consumption and weekly purchasing of fruits and vegetables and perception of overall dietary health.

Although people who live in areas with greater access to retailers of more healthful foods may have a better diet than those with limited access, dietary quality is still fairly low even in areas of high access (21). Interventions to test improving access alone versus improving access in conjunction with individual strategies that encourage people to choose more healthful foods are still needed. For these reasons, improving access to healthful foods in the community is only one of the mechanisms CPPW awardees are using to support more healthful dietary choices (6). For example, the Tennessee CPPW awardee assisted store owners with product placement to increase visibility of more healthful items and developed promotional posters, signage, and point-of-purchase flags that were posted in neighborhoods served by the stores and in the stores to increase awareness of the initiative. The awardee also provided samples of foods and beverages made with a variety of fruits and vegetables sold in the store to increase knowledge of different types of fruits and vegetables and how to prepare them.

Ensuring that all Americans have easy access to retail venues that offer affordable, healthful foods is an important step toward supporting more healthful choices and a high-quality diet in communities. Each of the 7 CPPW awardees highlighted in this commentary implemented an environmental change and has prospectively evaluated or plans to evaluate its effect. The 4 awardees improving access via farmers markets demonstrated that financial incentives increased sales at farmers markets; 2 of them also demonstrated that incentives increased fruit and vegetable intake. The 3 awardees improving access via corner stores each engaged the community to develop a context-specific approach to improving access and established a baseline. Postevaluation in each is under way. Each of the prospective program evaluations in these communities can help others understand how they can implement retail environment changes that may prompt changes in behavior. Continued evaluation efforts that prospectively evaluate retail environment changes like CPPW and stronger research study designs that can rule out alternative explanations for observed associations (22) can help to refine future investments in public health initiatives to improve diet and reduce the burden of nutrition-related diseases.
